# A Multivariate and Spatiotemporal Analysis of Water Quality in Code River, Indonesia

**DOI:** 10.1155/2020/8897029

**Published:** 2020-11-27

**Authors:** Mochamad A. Pratama, Yan D. Immanuel, Dwinanti R. Marthanty

**Affiliations:** Department of Civil and Environmental Engineering, Universitas Indonesia, Depok 16424, Indonesia

## Abstract

The efficacy of a water quality management strategy highly depends on the analysis of water quality data, which must be intensively analyzed from both spatial and temporal perspectives. This study aims to analyze spatial and temporal trends in water quality in Code River in Indonesia and correlate these with land use and land cover changes over a particular period. Water quality data consisting of 15 parameters and Landsat image data taken from 2011 to 2017 were collected and analyzed. We found that the concentrations of total dissolved solid, nitrite, nitrate, and zinc had increasing trends from upstream to downstream over time, whereas concentrations of parameter biological oxygen demand, cuprum, and fecal coliform consistently undermined water quality standards. This study also found that the proportion of natural vegetation land cover had a positive correlation with the quality of Code River's water, whereas agricultural land and built-up areas were the most sensitive to water pollution in the river. Moreover, the principal component analysis of water quality data suggested that organic matter, metals, and domestic wastewater were the most important factors for explaining the total variability of water quality in Code River. This study demonstrates the application of a GIS-based multivariate analysis to the interpretation of water quality monitoring data, which could aid watershed stakeholders in developing data-driven intervention strategies for improving the water quality in rivers and streams.

## 1. Introduction

Urbanization and rapid population growth are highly associated with the deterioration of surface water quality [1]. According to the Ministry of Environment and Forestry of Indonesia, of 619 monitoring stations across the country, 56% of them were classified as being heavily polluted [2]. Heavily polluted surface water is strongly associated with overburdened water treatment plants and decreased treatment efficiency, along with reduced drinking water quality, and increases in public health risks [3]. As surface water quality is dramatically affected by the change in land use and land cover (LULC), the deterioration of water quality in Indonesia is worsened by the rapid growth of the human population and the urban-rural expansions of built-up areas [4]. According to the latest report, the Indonesian population has been growing by about 1.1% annually, which corresponded to the annual expansion rate of urban settlement and deforestation rates of 7.2% and 0.5%, respectively [5, 6].

Understanding the relationships between LULC and water quality is important for watershed planning and management, and so the impact of nonpoint source pollution on water quality must be assessed [7, 8]. Runoff from catchment areas flowing into rivers carries land use-dependent contaminants, which affect the relationship between surface water quality and changes in LULC, and can be quantitatively correlated [9]. Various studies have been performed to correlate the two variables. One, by Hua [10], found that changes in built-up areas were strongly correlated with the variability of several water quality parameters, such as total coliform, biological oxygen demand (BOD), chemical oxygen demand (COD), total suspended solid (TSS), mercury, zinc, and iron. Rodriguez-Romero et al. [9] demonstrated the impact of LULC changes on the water quality of rivers flowing through cloud forests in tropical zones. In turn, a study by Huang et al. [11] suggested a significant positive correlation between the change in forest land and grassland and water quality, whereas changes in the built-up area had a significant negative correlation with water quality. GIS-based analysis, coupled with multivariate statistical analysis conducted by Bu et al. [12], indicated that regions dominated by agricultural and built-up areas in river watersheds tend to have lower water quality than other areas. Furthermore, important findings by Kändler et al. [13] suggest that built-up areas significantly affect the chemical composition of surface water if their proportion of land use is >20% of the total catchment area and if the proportion of cropped areas is <10%. In addition, rivers with watersheds that are >70% forested tend to have low nutrient and heavy metal concentrations [13].

Several studies have coupled the GIS-based analysis of LULC changes with multivariate statistical analyses in an effort to identify the main source of variability in surface water quality. Thuong et al. [14], for instance, identified the sources of lead, zinc, and cadmium contaminations in river sediment using cluster analysis (CA) and principal component analysis (PCA), whereas Chow and Yusop [15] employed CA and PCA for the identification of contamination sources in runoff water. In turn, a study by Lee et al. [16] used runoff quality data collected at the outlets of subcatchments with various LULC compositions in PCA in order to identify the sources of runoff contamination. The results extracted five principal components strongly associated with natural processes, agricultural activities, domestic wastewater, and urban areas.

Unfortunately, only a few studies have been conducted in Indonesia to analyze correlations between changes in the LULC and the parameters of surface water quality. A study conducted by a team from Bogor Agricultural University attempted to connect the water quality of Ciliwung River with changes in land use between 2010 and 2014 and found a strong positive correlation between the percentages of urban areas with total nitrogen and ammonia nitrogen concentrations [17]. Suharyo [18] also conducted a spatial analysis of the water quality of Opak River and found a positive correlation between the COD and urban areas. These two studies, however, did not establish significant statistical relationships due to the data having a relatively low temporal resolution. A study by Kuntoro et al. [19] demonstrated the impact of LULC changes on river discharge and found that the combination of anthropogenic activities and climate change may have caused significant decreases in the flow rate of the Upper Citarum River's watershed. Although highly temporally resolved data were used, the impact of changes in a specific LULC on a specific river quality parameter was not discussed.

In this study, a spatial and temporal trend analysis is performed on the water quality of Code River and changes in the LULC of the river's subcatchment areas for the period 2011–2017. The results were then used to analyze the LULC type that significantly influences certain water quality parameters. Finally, a multivariate analysis using PCA was performed on 11 water quality parameters collected between 2011 and 2017 at three monitoring stations in order to identify possible sources of contamination.

## 2. Materials and Methods

### 2.1. Study Location

Code River crosses three regencies/cities in Yogyakarta Province, Indonesia. It has a length of 41 km, starting from its upstream, which is located at Merapi Mountain (Sleman Regency), with its middle stream crossing through the densely populated areas and Yogyakarta City, to its downstream, which enters the Opak River System in the Bantul Regency. The watershed has a total area of ±58 km^2^ and is located in the three administrative areas of Sleman Regency, Yogyakarta City, and Bantul Regency. Most of the Code River watershed area in the Sleman Regency area corresponds to 71.44% of the total area of the Code River watershed.

The average annual temperature is around 24°C. September is the hottest month and has an average temperature of 27°C, whereas the coldest month is April, with an average temperature of around 20°C [20]. The average annual rainfall is 2802 mm. The month with the highest rainfall is January, with an average of 538 mm, and the lowest is September, with an average of 8 mm [21]. Code River plays an important role in the lives of the inhabitants of Yogyakarta. Its water is used for irrigation, as a drinking source, for fisheries, and other applications [22]. Along with the increases in population, Code River's watershed was designated for residential development. As a result, river water pollution, the narrowing of river bodies, high erosion levels, and sedimentation, to the point of frequent flooding in the Code River watershed, have been observed. In addition, as the river stream originates from an active volcano, the river often experiences floods caused by the fall or drift of cold lava settled in the dome of Mount Merapi, disturbed by rainfall in the area around the mountain.

**Figure 1:**
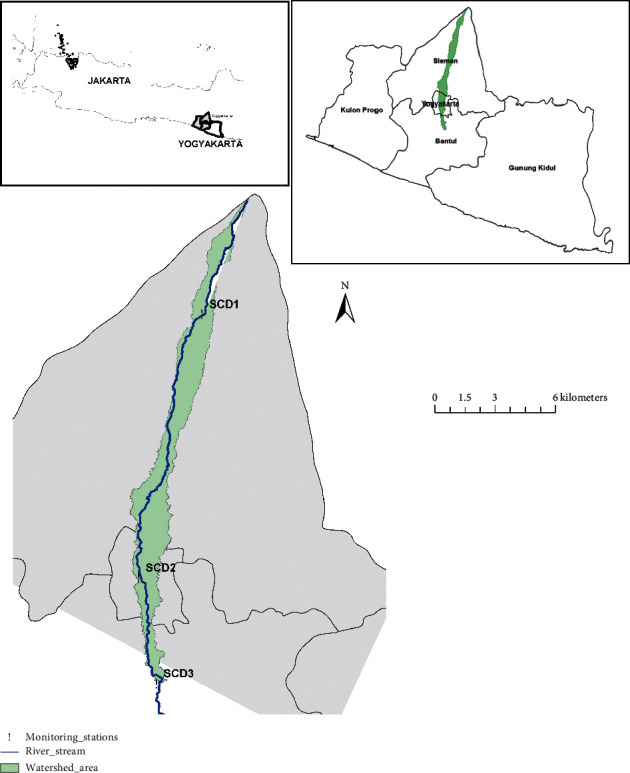
Code River watershed and water quality monitoring stations.

The Code River watershed, analyzed in this study, and was divided into three segments: the upstream watershed, starting from Mount Merapi and running to the SCD1 monitoring station; the middle stream watershed, starting from the SCD1 monitoring station and running to the SCD2 monitoring point in Yogyakarta City; and the downstream watershed, starting from the SCD2 monitoring station and terminating at SCD3 (Figure 1).

### 2.2. Water Quality and GIS Data

In this study, water quality data for Code River were provided by the Environmental Agency (BLH) D. I. Yogyakarta (DIY). These data were collected 2 to 3 times annually during the 2011–2017 period at the three monitoring stations mentioned (SCD1, SCD2, and SCD3). Each set of monitoring data consists of the following parameters: temperature (T), pH, total dissolved solid (TDS), TSS, dissolved oxygen (DO), BOD, COD, nitrate (NO_2_), nitrite (NO_3_), detergent (DET), phosphate (PO_4_), zinc (Zn), copper (Cu), lead (Pb), and fecal coliform (FC). The summary of the monitoring data is presented in Table 1.

Two types of satellite data were used in this study, the first being the Digital Elevation Model (DEM) image and Landsat images. The DEM images were used in the process of delineating Code River's watershed and were provided by the Indonesian Geospatial Information Agency, whereas the Landsat data were used in the land cover classification process for the entire study area and collected by the USGS.

### 2.3. LULC Classification

This LULC classification was conducted using the supervised classification method on the Landsat data. The maximum likelihood classification approach, using ArcGIS 10.3, was chosen, wherein each pixel is grouped to the class that has the highest similarity/probability based on the collected training samples [23]. After successful extraction, the LULCs were categorized according to the National Standard of Indonesia (SNI-7645-1-2014), which classifies LULC into six cover types, as described in Table 2.

### 2.4. Principal Component Analysis (PCA)

In this study, the two-step transformation data normalization method was used to transform the raw water quality data so that multivariate normal data could be obtained. The two-step transformation approach transforms any variable with a high number of levels and the effect of mode is negligible on the normality of statistical data [24]. We set principal components extracted from the analysis to have eigenvalues greater than 1 to be further analyzed. This study also used the varimax rotation method resulting in Varifactors (VFs), which provide information on the water quality parameters that are mostly responsible for the variability of water quality data. The validity of the PCA results was evaluated with the Kaiser–Meyer–Olkin (KMO) test and Bartlett's test of sphericity. High values close to 1.0 in the KMO test and small values (less than 0.05 of the significance level) in Bartlett's test indicate that PCA might be useful to apply to the water quality data [25–27].

## 3. Results and Discussion

### 3.1. Spatial Trend of Code River Water Quality

**Figure 2:**
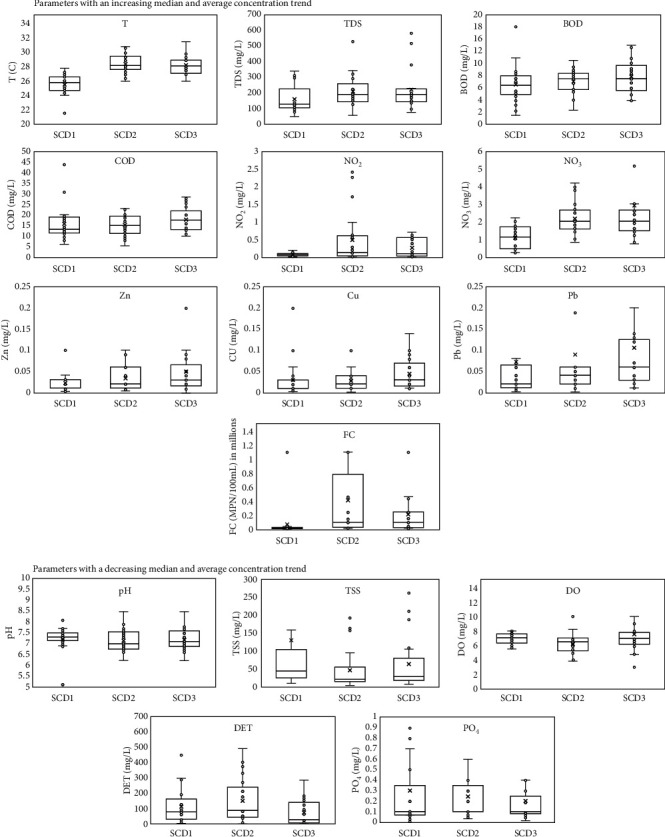
The spatial trend of water quality in Code River.

In general, parameters of water quality of the Code River can be grouped into (a) parameters with an increasing median and average concentration trend as the Code River flows from upstream to downstream: T, TDS, BOD, COD, NO_2_, NO_3_, Zn, Cu, Pb, and FC; (b) parameters with a decreasing median and average concentration trend as the Code River flows from upstream to downstream: pH, TSS, DO, DET, and PO_4_ (Figure 2). It can be seen in Figure 2 that the mean of water temperature increases by more than 2°C from SCD1 to SCD2 and SCD3. The surface water temperature is greatly influenced by the ambient temperature and sun cover [28]. The temperature increase is to be expected, because SCD2 and SCD3 are located in city areas where the ambient temperature is higher. Intensive anthropogenic activities can also increase ambient temperatures in urban areas [29]. In addition, Kinouchi et al. [30] found that urban domestic wastewater is also a major factor in increasing river water temperature over long periods.

In general, there were fluctuations in the TSS concentrations along Code River, with the highest median TSS concentrations at the SCD1 monitoring point, which then decreased as it entered SCD2, before increasing again around SCD3. Several factors affect TSS concentrations in surface water, including water discharge and weather, the presence of industrial activities, domestic and agricultural runoff, and LULC changes that cause soil exposure, resulting in erosion [31]. The same phenomenon was also observed for NO_2_, NO_3_, BOD, and COD as the concentration trends increased from SCD1 to SCD3. The spatial trend for the PO_4_ median concentrations revealed an insignificant difference between SCD1, SCD2, and SCD 3, which was 0.1 mg/L. It should be noted, however, that if the outliers at all of the points are neglected, the highest average PO_4_ concentration will be in the river's middle stream component. The location may receive a large amount of PO_4_ from agricultural runoff and domestic wastewater, which in turn increases PO_4_ concentrations in the water body.

Increases in the trend of Zn, Cu, and Pb concentrations starting from the upstream point to the middle stream and ending in the downstream area were also observed. Zn contamination in bodies of water primarily results from anthropogenic activities in the vicinity of rivers [32]. Nugraha [33] suggested that the main contributor to metal pollution in rivers is industrial and domestic wastes along the river basin. Therefore, it was expected that metal concentrations would increase as the river flowed downstream.

The spatial trend shows that FC concentrations in Code River are fairly alarming. Despite SCD1 being located in the upstream area, even the lowest concentration of FC did not meet the water quality standards set out by the provincial government. Before entering the middle stream section, Code River flows through a large area of agricultural land and densely populated urban areas. Consequently, a dramatic jump in the FC concentrations in the middle stream area was observed. According to Rompré et al. [34], the large populations of FC bacteria are strongly influenced by human activity. The presence of these FC bacteria almost always indicates water contamination by feces, from either humans or other mammals [35]. At the SCD3 monitoring point, which is downstream, there was a decrease in the average concentration of FC, although the median tended to be constant. This may be explained by the dilution process in the river, which results in decreases in the average FC concentration. Fajri et al. [36] suggest that Code River's water quality in the downstream section was better because of the high discharge from the catchment area, which reduced the concentrations through dilution.

### 3.2. Temporal Trend of Code River's Water Quality

**Figure 3:**
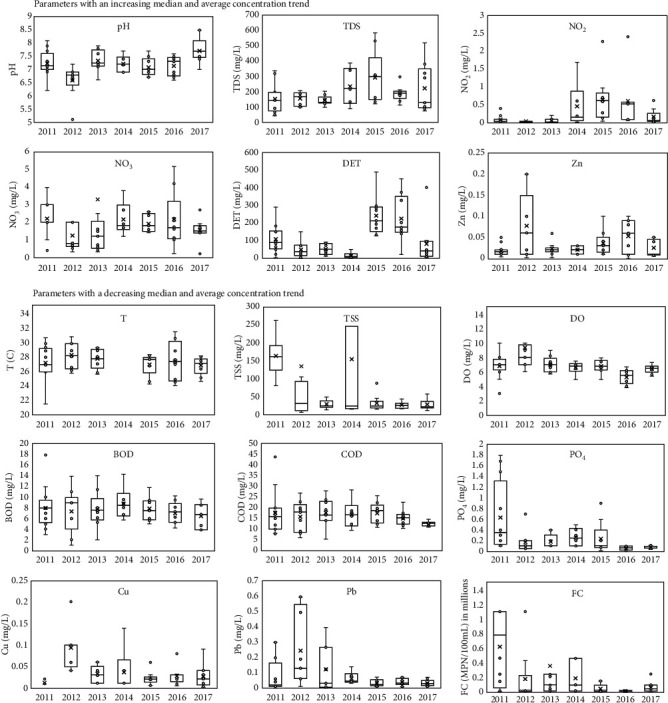
Temporal trend of the water quality in Code River.

On the basis of the temporal trend analysis from 2011 to 2017, the water quality parameters could be grouped into (a) parameters with an increasing concentration trend, such as pH, TDS, NO_2_, NO_3_, DET, and Zn, and (b) those with a decreasing concentration trend during the monitoring period, such as T, TSS, DO, BOD, COD, PO_4_, Cu, Pb, and FC (Figure 3).

As is shown in Figure 3, the temperatures of Code River recorded each year fluctuated and had an overall decreasing trend. The median temperature value per year was in the range of 26.9°C to 28.1°C. The lowest water temperature recorded (21.5°C) was measured in 2011, whereas the highest water temperature recorded was 31.4°C in 2016. The fluctuations in water temperature were greatly influenced by local weather conditions during the time of operation. Figure 3 also shows an extreme period of TSS concentrations in 2011 and 2014, which were significantly longer compared with those of other monitoring years. This may be due to the occurrence of cold lava floods that carried the remnants of Mount Merapi's eruption material in 2011 [37]. In addition, the extensive flooding that occurred may also have increased soil erosion in the river bodies, resulting in high TSS concentrations in 2011.

Significant trends of BOD and COD concentrations during 2011–2017 were not observed. With that being said, the concentrations consistently conformed to the lowest water quality standards set by the provincial government. This may indicate that there has been considerable and consistent loading of domestic wastewater into Code River. Organic materials present in river bodies usually originate from the decomposition of dead animals and plants, as well as domestic and industrial wastewater. Astari [38] suggested that the increase in the concentration of BOD in the middle stream section of Code River (SCD2) was caused by increases in domestic waste loading from settlements or communal sewage treatment plants. Moreover, most residents along the banks of Code River use the river as a receiving water body for discharges of domestic wastewater. These domestic activities could be associated with organic and nutrient contamination, which is commonly detected in the greywater of domestic waste.

Decreasing trends of Pb and Cu concentrations during 2011–2017, with a dramatic increase in 2012, were also observed. The presence of Pb and Cu in water bodies could have originated from natural or anthropogenic activities. As the river originated in a volcano, the metals contained in the mountain's rock or soil layers could be easily washed off during rainfall and then enter the river bodies. In general, all metal concentrations measured at the three monitoring stations still conformed to the highest quality standards set by the provincial government, with the exception of the extreme values detected in 2012.

The concentrations of FC in Code River from 2011–2017 fluctuated with a decreasing trend. The lowest FC concentration was 3,000 MPN/100 mL, detected in June 2015, whereas the highest was 2,300,000 MPN/100 mL in February 2013. Anthropogenic waste can be a source of disease, as blackwater from domestic activities transport pathogens such as *Escherichia coli*, *Vibrio cholera*, *Shigella* sp., *Campylobacter jejuni*, and *Salmonella*, which are categorized as FC bacteria [39]. The presence of these bacteria in Code River is quite alarming, given that their concentrations at all monitoring points are critically below the lowest water quality standards. Moreover, exposure to these bacteria directly increases health risks to inhabitants in Code River's catchment area.

### 3.3. LULC Changes

**Figure 4:**
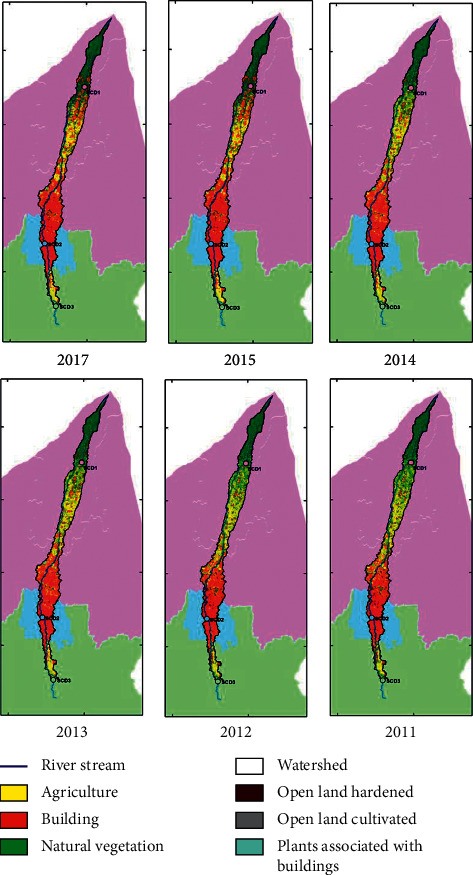
LULC classification of the code river watershed in 2011–2017.

The LULC classification was carried out for the same period as the water quality monitoring period of 2011–2017. However, the classification for 2016 was not conducted because of the high percentage of cloud cover in the Landsat image during that year.  Figure 4 shows the results of the LULC classification for 2011–2017 (except for 2016) for the entire Code River watershed.

In general, the natural vegetation class (VA) saw a negative trend in the period 2011–2017. In 2011, it occupied 24% of the watershed area, whereas by 2017, the percentage had dropped to 21% (Table 3). The agricultural land cover class (AG) also decreased over the period at an average annual rate of 5% of the total area of the Code River catchment. On the other hand, the land cover class of building areas (AB), the plants associated with buildings (TB), and the cultivated and hardened open land had an increasing trend, with average annual growth rates of 4%, 9%, and 162%, respectively. The significant growth of TB may be associated with the recent trends for parks and green roof spaces in urban areas. The trends of the three classes may correspond to population growth and expansions in the urban areas. A strong trend of the natural/seminatural class was not observed, indicating that the least development was in the area on Merapi Mount, which is likely because it erupts relatively often.

### 3.4. Correlation Analysis between the LULC Changes and Water Quality

The correlation between the changes in the LUCC and water quality was quantified by means of a Pearson correlation coefficient. The annual changes in each LULC class in each sub-watershed (Upper, Middle, and Lower) were cross-correlated with each water quality parameter (Table 4). In the upper watershed, no significant correlation was observed between VA and any other water quality parameter. This may be because annual changes in WA were very small. T, pH, and BOD were found to have strong associations with AB (−0.858, 0.871, and 0.845, respectively). This was expected, as wastewater from domestic activities in AB contains high BOD concentrations and is slightly alkaline [40]. The same phenomenon was also observed in the middle stream area, where strong and significant correlations between AB and T, pH, and BOD were obtained (−0.834, 0.756, and 0.8, respectively).

A high correlation between the AG and NO_3_ (0.904) was found in the upper watershed. Nutrients such as NO_3_ are highly associated with agricultural activities due to the widespread use of nitrogen-based fertilizers [41]. In the lower watershed, VA had a significant negative correlation with NO_2_ and DET (−0.798 and −0.734, respectively). The two parameters are commonly found in greywater. As the lower watershed is characterized by a highly densely populated area, a strong negative correlation between natural areas and nutrient contaminants was obtained. Nutrient pollutants such as NO_2_, NO_3_, and DET are the most pervasive contributors to poor water quality in the world [42]. With that being said, a statistically significant correlation between the BOD and AB or VA was not observed. This may be due to the dilution process in the lower watershed, as was mentioned in the previous section.

Strong positive correlations between the Cu, Pb, and VA were unexpectedly obtained (0.787 and 0.817, respectively). The main source of the Cu and Pb in the atmosphere is fuels [43]. During combustion in an engine, metals can be emitted to the atmosphere through the exhaust and settle in open spaces. The settled metals, thereafter, are washed away to water bodies by runoff during rain events. It should be noted, however, that the obtained strong correlations could have no association. Further studies are therefore required to clarify correlations between heavy metals and VA.

### 3.5. Pollution Source Identification

Sources of Code River contamination were identified on the basis of the PCA results. Preliminary tests, including the KMO, Bartlet test, anti-image test, and scree plot, were conducted prior to the analysis in order to determine whether the water quality data conformed to the statistical criteria. On the basis of the results of these tests, pH, NO_3_, and ZN were not included in the PCA, as the MSA values of the anti-image test were over 0.5 [44]. Moreover, DET was also excluded from the analysis because its value of commonalities was over 0.5 [45]. Thus, the remaining water quality parameters included in the PCA were T, TDS, TSS, DO, BOD, COD, NO_2_, PO_4_, Cu, Pb, and FC.  Table 5 shows the results of the PCA that extracted four rotated principal components (VFs) and significant loadings from the water quality parameters to each PC.

Table 5 shows the VFs extracted from the water quality data, which explain 69% of the total data variance. The VF1 explains 23.18% of the total variance in the water quality data and has a very strong positive loading in BOD and COD. The VF1 may represent organic matter pollutants in Code River. According to a study by Hua [10], a PCA in Mallaca River extracted the main VF and had a positive loading effect on the BOD and COD. The main component was associated with the natural decomposition process and anthropogenic domestic waste. VF2 corresponds to 17.81% of the total variance in the water quality data and had a positive loading effect on FC, PO_4_, T, and TSS. PC2 may represent sources of nutrient pollutants and FC bacteria in Code River, such as from agricultural runoff, domestic greywater, and blackwater. Agricultural runoff that flows into surface water usually contains high levels of nutrients (e.g., phosphorus and nitrogen), biologically degraded organic carbon, pesticide residues, and FC batteries (indicating contamination by animal waste) [46]. In addition to agricultural runoff, PO_4_ and TSS are also strongly present in domestic greywater, whereas FC and TSS are mostly found in domestic blackwater [47–49].

VF3 accounts for 15.467% of the total variance in the water quality data and has a positive loading effect on Cu, Pb, and T. PC3 might represent metal pollutants in Code River that may have originated from anthropogenic activity, such as urban runoff, domestic waste, or industrial waste. VF4, accounting for 12.464% of the total variance of water quality data, has a positive loading effect on DO, NO_2_, and TDS. The negative loading effect on DO indicates a negative correlation between water contamination and concentrations of DO. On the basis of the four VFs that affect the overall variability of Code River's water quality data, it was found that water quality parameters relating to domestic waste primarily affect the variability of Code River's water quality data.

**Figure 5:**
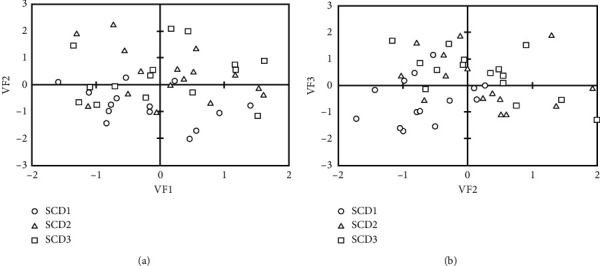
Scatter plot of scores for the four PCs obtained from each monitoring station.

For each observation in each monitoring station, a score was calculated using a linear regression equation with a constant, obtained from Table 5, for each water quality parameter. Thereafter, the scores were plotted as shown in Figure 4. In the first plot (Figure 5(a)), the scores of VF1 (representing organic pollution) from each observation were coupled with those of VF2 (representing domestic wastewater). As can be seen in Figures 5(a) and 5(b), the scores of SCD1 (circle) were scattered along the *x*-axis but were concentrated on the negative value of the *y*-axis. On the other hand, the scores of SCD2 (triangle) and SCD3 (square) were scattered along the *x*- and *y*-axes. This indicates that SCD1 was highly influenced by organic pollution, whereas SCD2 and SCD3 were impacted by both organic pollution and domestic wastewater. This could be explained by the low proportion of AB in the upper watershed compared with that in the middle and lower watersheds. Consequently, the influence of domestic wastewater was not observed in the VF2 scores of SCD2.

In Figure 5 on the right, the scores of VF2 (representing domestic wastewater) from each observation were coupled with those of VF3 (representing metal pollutants). The scores of SCD1 were mostly scattered on the negative quadrant of both VF2 and VF3, indicating that the influence of both sources of pollution on the variability of water quality data in SCD1 was insignificant. Moreover, high scores of VF2 and VF3 were obtained from the monitoring data from the SCD2 and SCD3 stations, indicating the significant influence of domestic and metal contaminants on water quality in the middle and lower streams. The findings were consistent with the results of the impacts of the LULC changes, which established significant correlations between the LULC changes in the middle and lower watersheds and the BOD, Cu, and Pb parameters.

## 4. Conclusions

This study demonstrated the use of GIS-based multivariate spatiotemporal analysis in explaining the variability of water quality in Code River, Indonesia, and also identified the main possible sources of water pollution in the river. Some water quality parameters have both temporally and spatially increasing trends, such as TDS, NO_2_, NO_3_, and Zn, which indicate that more concerns must be addressed with respect to the sources of these parameters. This study also found that the pollution levels of some parameters, such as BOD, Cu, and FC, were fairly severe and exceeded water quality standards.

On the basis of the correlation analysis between the LULC changes and water quality, the study found that land cover by natural vegetation has a positive impact on Code River's water quality, whereas agricultural land and building areas tend to negatively influence the overall water quality. Moreover, the PCA of the water quality data suggests that organic matter, metals, and domestic wastewater are the most prominent sources in explaining the total variability of the river's water quality. The results of this study can be used as a basis of water quality management in Code River's watershed and assist stakeholders in the selection of intervention methods for controlling wastewater pollution.

## Figures and Tables

**Table 1:** Summary of water quality in Code River from in three monitoring stations 2011–2017.

Parameter	T	pH	TDS	TSS	DO	BOD	COD	NO_3_	NO_2_	DET	PO_4_	Zn	Cu	Pb	FC
Unit	°C	mg/L	mg/L	mg/L	mg/L	mg/L	mg/L	mg/L	mg/L	mg/L	mg/L	mg/L	mg/L	MPN/100 mL	
Mean	27.31	7.19	195.02	80.75	7.21	7.64	16.31	2.07	0.27	112.74	0.25	0.04	0.03	0.09	230,460
Standard error	0.254	0.069	14.674	19.144	0.429	0.392	0.837	0.328	0.061	15.013	0.047	0.005	0.005	0.017	55,652
Median	27.5	7.2	167	28	6.8	7.6	15	1.7	0.06	75	0.1	0.02	0.02	0.04	23,000
Standard deviation	1.902	0.540	115.546	151.954	3.402	3.113	6.642	2.602	0.485	119.160	0.371	0.041	0.037	0.136	441,725
Kurtosis	0.571	3.087	2.518	20.345	25.297	1.345	3.804	45.620	10.580	1.650	8.963	6.795	5.743	6.104	9.102
Skewness	−0.314	−0.541	1.551	4.312	4.606	0.627	1.417	6.307	3.116	1.446	2.950	2.394	2.186	2.520	2.794
Range	9.9	3.4	546	903	25	17	38.7	20.7	2.428	491.8	1.8	0.2	0.199	0.599	2,397,000
Minimum	21.5	5.1	42	4	3	1	5.3	0.2	0.002	0.1	0	0	0.001	0.001	3,000
Maximum	31.4	8.5	588	907	28	18	44	20.9	2.43	491.9	1.8	0.2	0.2	0.6	2,400,000

**Table 2:** LULC classification.

LULC type	Code
Natural/seminatural vegetation	VA
Agriculture	AG
Open land cultivated/hardened surface	LP
Plants associated with buildings	TB
Building area	AB
Natural/seminatural open land	LA

**Table 3:** Proportions of each LULC class in the Code River watershed during 2011–2017.

Year	Percentage of LULC (%)					
	VA	AG	LP	TB	AB	LA
2011	24.46	32.72	2.27	0.04	36.91	3.07
2012	26.46	30.68	2.46	0.16	37.5	2.74
2013	23.79	29.51	2.12	0.86	40.34	3.39
2014	22.91	28.75	3.34	0.78	41.5	2.24
2015	22.21	28.16	2.66	1.31	42.74	2.92
2017	21.72	21.01	3.42	1.62	48.73	3.5

**Table 4:** Pearson correlation coefficients between the LULC and water quality parameters.

Parameter	Upper watershed			Middle watershed			Lower watershed		
	VA	AB	AG	VA	AB	AG	VA	AB	AG
**T**	0.398	−0**.858**^*∗*^	0.590	0.742	−0**.834**^*∗*^	0.685	0.502	−0.552	0.340
**pH**	−0.533	**0.871** ^*∗*^	−0.232	−0.346	**0.756** ^*∗*^	−0.702	−0.037	**0.955** ^*∗*^	−0**.782**^*∗*^
**TDS**	0.374	0.369	0.426	−0.207	0.438	−0.519	−0.655	0.608	−0.527
**TSS**	0.513	−0.473	0.045	−0.201	−0.476	0.507	0.580	−0.400	0.279
**DO**	0.336	−0.619	−0.227	0.720	−0.476	0.415	0.090	−0.508	0.328
**BOD**	0.022	**0.845** ^*∗*^	0.093	−0.054	**0.800** ^*∗*^	−0**.891**^*∗*^	0.313	−0.632	−0.067
**COD**	0.141	−0.533	0.574	−0.140	−0.199	0.219	0.411	−0.514	−0.037
**NO**_**3**_	−0.136	−0.028	**0.904** ^*∗*^	−0.383	−0.191	0.135	0.233	0.173	0.050
**NO**_**2**_	0.023	0.181	0.428	−0.474	0.202	−0.164	−0**.798**^*∗*^	0.020	0.077
**DET**	0.346	0.321	−0.394	−0.564	0.388	−0.214	−0**.734**^*∗*^	−0.143	0.602
**PO**_**4**_	−0.049	−0.529	0.650	−0.513	−0.499	0.588	0.588	−0.266	0.082
**Zn**	0.141	−0.048	−0.521	0.655	−0.231	0.183	−0.019	−0.587	0.431
**Cu**	0.271	−0.457	−0.416	**0.787** ^*∗*^	−0.488	0.399	0.047	−0.224	−0.320
**Pb**	0.350	−0.502	−0.272	**0.817** ^*∗*^	−0.635	0.498	0.316	−0.559	0.354
**FC**	−0.502	−0.626	0.310	−0.080	−0.584	0.646	0.635	−0.409	0.275

^*∗*^Statistically significant.

**Table 5:** Four extracted VFs from the 10 parameters of water quality.

Parameter	VF1	VF2	VF3	VF4
BOD	0.927			
COD	0.897			
FC		0.816		
PO_4_		0.727		
T		0.630	0.531	
TSS		0.514		
Cu			0.821	
Pb			0.751	
NO_2_				0.789
DO				−0.664
TDS				0.541

Eigenvalue	2.550	1.959	1.701	1.371
% variance explained	23.182	17.810	15.467	12.464

## Data Availability

The water quality and land use land cover data used to support the findings of this study are available from the corresponding author upon request.
